# Antibacterial mouthwash alters gut microbiome, reducing nutrient absorption and fat accumulation in Western diet-fed mice

**DOI:** 10.1038/s41598-024-54068-y

**Published:** 2024-02-18

**Authors:** Lucas Rannier Ribeiro Antonino Carvalho, Ariela M. Boeder, Miho Shimari, Andrei L. Kleschyov, Anders Esberg, Ingegerd Johansson, Eddie Weitzberg, Jon O. Lundberg, Mattias Carlstrom

**Affiliations:** 1 Department of Physiology and Pharmacology, Karolinska Institutet, Biomedicum, 5B, 17165 Solna, Stockholm, Sweden; 2 Department of Pharmacology, Federal University of Santa Catarina, Florianópolis, Brazil; 3 Department of Odontology, Umeå University, Umeå, Sweden; 4 Department of Perioperative Medicine and Intensive Care, Karolinska Hospital, Stockholm, Sweden

**Keywords:** Bacteria, Microbial communities, Gastroenterology, Diabetes, Metabolic syndrome, Obesity, Pre-diabetes, Cardiovascular diseases, Metabolic disorders, Experimental models of disease, Cardiovascular biology, Metabolism

## Abstract

Prolonged use of antibacterial mouthwash is linked to an increased risk of systemic disease. We aimed to investigate if disturbing the oral microbiota would impact the lower gut microbiome with functional effects in diet-induced obesity. Mice were exposed to oral chlorhexidine and fed a Western diet (WD). Food intake and weight gain were monitored, and metabolic function, blood pressure, and microbiota were analyzed. Chlorhexidine reduced the number of viable bacteria in the mouth and lowered species richness in the gut but with proportional enrichment of some bacteria linked to metabolic pathways. In mice fed a Western diet, chlorhexidine reduced weight gain, body fat, steatosis, and plasma insulin without changing caloric intake, while increasing colon triglycerides and proteins, suggesting reduced absorption of these nutrients. The mechanisms behind these effects as well as the link between the oral microbiome and small intestinal function need to be pinpointed. While the short-term effects of chlorhexidine in this model appear beneficial, potential long-term disruptions in the oral and gut microbiota and possible malabsorption should be considered.

## Introduction

The oral and gut microbiota, comprising trillions of bacteria and other microorganisms, plays crucial roles in maintaining human health^1^. Accumulating research shows that disturbances in microbiota content and diversity may contribute to the development and progression of cardiometabolic diseases, including cardiovascular disease, obesity, type 2 diabetes, and metabolic syndrome^2–5^.

The gut and oral microbiomes are the largest microbial ecosystems in the human body. The human microbiome project, initiated in 2007^1^, revealed that over half of the body's bacteria reside in the lower gastrointestinal (GI) tract (29%) and the oral cavity (26%)^6,7^. These environments are ecologically rich and diverse but separated by barriers like physical distance, gastric acid and bile^6,7^. Nevertheless, disruption of these barriers, as observed in neonates or the elderly, can facilitate communication between these regions. Additionally, it has been demonstrated that oral microbes can breach these barriers under specific conditions and potentially transfer to the gut, establishing a dynamic interaction known as the oral-gut microbiome axis^8^.

Separately, the oral cavity harbors a diverse microbial community with approximately 700 bacterial species or phylotypes^9^. Microorganisms are divided into oral ecological niches, i.e., saliva, tongue, gingiva, buccal mucosa, palate, and dental subgingival/supragingival sites, with variations in taxonomic profiles and microbiota activity^10^. The oral microbiota has a key role in nitric oxide (NO) biology, through the reduction of salivary nitrate to nitrite in the mouth. Nitrite can then become bioactive NO throughout the body in a system known as the nitrate-nitrite-NO pathway. This process influences various functions, including metabolic and cardiovascular health^11,12^. Conversely, poor oral hygiene, periodontal disease (gum disease), and dysbiosis (microbial imbalance) in the oral microbiota have been linked to systemic inflammation and an increased risk of cardiometabolic diseases^8,13^.

The gut microbiota refers to the microbial community residing in the lower GI tract, the largest and the most well-characterized microbial ecosystem in the human body^14^. A healthy and diverse gut microbiota is essential for proper digestion, nutrient absorption, immune function, and maintenance of gut barrier integrity^15^ and disruptions in gut microbiota can impact cardiometabolic health through various mechanisms, including metabolic regulation, gut barrier maintenance, and immune system modulation. Gut bacteria metabolize dietary components, producing beneficial metabolites like short-chain fatty acids (SCFAs) that reduce inflammation and support metabolism^16^. A balanced gut microbiota also helps to protect the intestinal barrier, preventing harmful substances from entering the bloodstream. Dysbiosis, however, can compromise this barrier, allowing toxins and metabolites to contribute to inflammation and metabolic issues^17,18^. Furthermore, gut bacteria influence the immune system, affecting systemic inflammation^19,20^. Imbalances in the gut microbiota can lead to immune dysfunction and a pro-inflammatory state linked to cardiometabolic diseases^5^.

From a functional point of view, it is known that oral and gut microbiomes interdependently regulate physiological functions and pathological processes, and through the microbial transmission axis can shape and/or reshape the microbial ecosystem in both habitats, eventually modulating disease pathogenesis^21^. However, the oral-gut microbiome crosstalk, the adaptive characteristics of microbiomes facing different dietary challenges, and their pathophysiological influence are less explored. Although, it is well-documented that the use of antiseptic mouthwash with chlorhexidine significantly affects the oral microbiota less is known on how this may influence gut bacteria in health and disease. The aim of this study was to evaluate the effects of oral microbiota disturbance by a chlorhexidine-based antibacterial agent on the intestinal microbiota, nutrient absorption, and obesity induced by a high-fat, high-sugar diet. We hypothesized that disruption of commensal oral bacteria by chronic exposure to antiseptic mouthwash may influence gut microbiota content and diversity, and therefore modulate the phenotypical response to a dietary challenge with Western diet.

## Materials and methods

### Animals and experimental design

The study is reported in accordance with ARRIVE guidelines and was approved (ID: 17128-2021) by the Regional Institutional Animal Care and Use Committee at Karolinska Institutet in Stockholm, Sweden and was performed according to the NIH guidelines and with the EU Directive 2010/63/EU for the conduct of experiments in animals. Male mice (C57BL/6J) were obtained from Janvier Labs (Le Genest-Saint-Isle, France) and housed in conditions of controlled temperature, humidity, and light-and-dark cycle (12/12 h), with ad libitum access to food and water.

The study was conducted in two rounds of experiments according to the different diets used. For the first round, twenty-four animals were fed standard rodent chow (RD) (CRM(P) 801,722, SAFE, Rosenberg, Germany) and randomly divided into two groups (12 mice in each group); one was exposed to mouthwash with a commercially available chlorhexidine solution (0.2%, Corsodyl, Stockholm, Sweden) for 8 weeks and the other with a saline solution (control).

In the second round, with a different batch of animals, the same experimental design and group sizes were used (i.e., chlorhexidine mouthwash vs. saline) but with animals fed a Western diet (WD) with a high fat and sugar content, obtained from Research Diets Inc. (New Brunswick, NJ, USA) for 8 weeks.

Exposure to chlorhexidine mouthwash was performed with a swab and the solution was distributed directly into the oral cavity of the mice. This procedure was performed three times *per* week, for a total period of 8 weeks. Treatment frequency, as well as concentrations and methods used for the mouthwash to cause a disturbance in the oral microbiota were evaluated by prior pilot experiments (Supplementary material—Figures S1–S2). After baseline data collection and the beginning of the dietary and mouthwash interventions, the animals were evaluated weekly with weight gain and water/food intake recordings.

### Body composition

At the end of the experimental period, body composition was quantified by dual-energy X-ray absorptiometry (DEXA), using a Medikors InAnlyzer densitometer (MEDIKORS Inc., Seongnam, Republic of Korea). Fat and lean masses were calculated in relation to body weight.

### Metabolic parameters

The metabolic parameters evaluated in vivo were plasma glucose concentrations in fasting (5-h) and non-fasting conditions, and intraperitoneal glucose (ipGTT) and insulin (ipITT) tolerance tests, as previously described^22^. Blood glucose levels were monitored by FreeStyle Lite Blood Glucose Meter (Abbott Diabetes Care Inc, Alameda, CA, USA). For ipGTT, the mice were fasted for 5 h at the same time of day (test started at approximately 1 p.m.). The mice were injected with 50% D-glucose solution (2 g/kg body weight) and blood glucose was evaluated at t = 0, 15, 30, 60, and 120 min after glucose administration. For ipITT, the procedure was similar to the ipGTT but the mice were not fasted. In the morning, mice were injected with insulin (0.75 IU/kg body weight; Novorapid 100 IU/ml, Novo Nordisk A/S, Bagsvaerd, Denmark), using a 0.25 IU/ml solution, and blood glucose measurements were taken repeatedly at the same timepoints as for the ipGTT.

### Blood pressure

Coda High Throughput Noninvasive Tail Monitoring System (Kent Scientific, Torrington, CT, USA) was used for conscious blood pressure monitoring, following the manufacturer's protocol, as previously described^22^. A 3-day training period was used to minimize the degree of stress whereafter systolic (SAP), diastolic (DAP), and mean blood pressure (MAP) were quantified by 3 cycles of 15 repetitions. Averaged data from each animal were used for analysis. Blood pressure assessment was performed before the other in vivo tests to avoid interference and reduce stress.

### Biochemical analysis

#### Plasma insulin

Insulin was quantified by the Mouse Insulin ELISA Kit (No. 10-1247-10; Mercodia AB, Uppsala, Sweden). This analysis used 5 µL of plasma according to the manufacturer's instructions. The assay range was 0.2–6.5 µg/L and the limit of detection was ≤ 0.2 µg/L. Both intra-assay and inter-assay coefficients of variation were ≤ 10%.

#### Nitrate, nitrite, and heme-NO measurement

Plasma and urinary levels of nitrate and nitrite were analyzed by HPLC (ENO-20) as described previously^23^. In brief, samples (10 μl) were injected using a Hamilton syringe, and nitrite and nitrate were separated by reverse phase/ion exchange chromatography followed by nitrate reduction to nitrite by cadmium and reduced copper. Griess reagent was then used to derivatize nitrite to form diazo compounds and analyzed (detection at 540 nm). Values and concentrations were corrected when it was necessary to dilute the urine.

Red blood cell (RBC) heme-NO levels were evaluated by Electron Paramagnetic Resonance (EPR) using an X-band table-top spectrometer MS5000 (Bruker-Magnettech, Germany). The EPR spectra were recorded at 77K and the instrument parameters were 10 mW microwave power, 0.6 mT amplitude modulation, 100 kHz modulation frequency, 330 mT center field, 40 mT sweep width, 60 s sweep time and 4 scans. The RBC heme-NO levels were assessed by measurement of the first component of the heme-NO triplet EPR signal (g-factor = 2.01; A_N_ = 1,7 mT)**.** EPR data were expressed in arbitrary units (a.u.).

#### Triglycerides, cholesterol and total protein

To quantify triglycerides and cholesterol in plasma and fecal samples, the Triglyceride Colorimetric Assay Kit (No. 10010303; Cayman Chemical, Michigan, USA) and the Cholesterol Fluorometric Assay Kit (No. 10007640—Cayman Chemical, Michigan, USA) were used, respectively. Samples were collected in a fed state and at the same time of day for all groups, the procedures and dilutions were performed according to the manufacturer's instructions. Total protein in feces and colon contents was determined by colorimetric method using the Bio-Rad's protein micro assay (No. 5000006) and Bradford assay from Sigma-Aldrich (No. B6916; Sigma–Aldrich, St Louis, Missouri, USA) in fresh samples collected in fed state, homogenized with bullet blender.

#### Lipase activity

Tongue lipase activity was measured in the tongue tissue sample using a commercially available kit from Sigma-Aldrich (No. MAK048; Sigma–Aldrich, St Louis, Missouri, USA) based on a coupled enzymatic reaction using methylresorufin as a standard following the manufacturer's instructions. The value was corrected by the protein concentration in the homogenized tongue tissue sample.

### Microbiome analyses

#### Total bacteria count

To measure total bacterial count at the end of the 8-week period, samples were collected from the oral cavity and cecal contents with a sterile swab and uniformly distributed on blood agar plates. The plates were incubated for 18 h in an aerobic environment, and the colony forming units (CFU) were counted from the photographic scan using the ImageJ Software^24^.

#### Bacteria 16S rRNA gene amplicon sequencing, processing, and taxonomic assignment

DNA was extracted using the DNeasy PowerSoil Pro Kit (QIAGEN, Kista, Sweden) with 10 min maximum speed vortexing in a Vortex Adapter (QIAGEN) using 10–15 mg mouse feces. The DNA quality was estimated using a NanoDrop 1000 Spectrophotometer (Thermo Fisher Scientific, Uppsala, Sweden) and the quantity by the Qubit 4 Fluorometer (Invitrogen, Thermo Fisher Scientific, Waltham, MA, USA).

The v3-v4 16S rRNA gene segment was amplified (KAPA HiFi HotStart ReadyMix (2×), Wilmington, MA, USA) by PCR (denaturing at 98 °C for 3 min; 30 cycles with denaturing at 94 °C for 20 s, annealing at 51 °C for 20 s, and extension at 72 °C for 20 s; followed by 10 min at 72 °C; and 4 °C to finish). The 341F (CCTACGGGNGGCWGCAG) forward and 806R (GGACTACHVGGGTWTCTAAT) reverse primers containing a linker sequence, a 12 bp barcode, and the Illumina adapter were used as described by Caporaso et al.^25^ Purified equimolar amplicons (pool of all samples) adjusted to 4 nM, spiked with 5% PhiX (Illumina, Eindhoven, the Netherlands), denatured, and diluted according to Illumina instructions were loaded and sequenced using MiSeq cartridges (Illumina, San Diego, CA) at the Swedish Defense Research Agency research facility in Umeå, Sweden. The generated raw v3–v4 amplicon sequences were demultiplexed using deML^26^, paired-end reads were merged, and primers and ambiguous and chimeric sequences were removed using default settings in DADA2 within QIIME2^27^ with the resolution of amplicon sequence variants (ASVs). Taxonomy was assigned to the ASVs using the SILVA 132_99_nb_classifier inside QIIME2. ASVs present in ≥ 2 animals and at ≥ 97% identity with a named species/unnamed phylotype were retained, and those with the same taxonomic identity were aggregated.

The microbiota diversity was evaluated using α-diversity and β-diversity. The Evenness and Shannon diversity indexes were used to assess α-diversity. Bray Curtis, Jaccard, unweighted Unifrac, and weighted Unifrac distance were used to evaluate β-diversity. The FDR-derived q-value is reported as the adjusted *p*-value for diversity measurements.

### Tissue collection and histological evaluation

Tissues (intestinal, liver, and fat) were collected, immediately weighed, frozen in liquid nitrogen or fixed in 10% formalin solution for histopathological evaluation. After fixation, the samples were embedded in paraffin and cut using a microtome (5 µm). The slides were stained with hematoxylin–eosin and blindly evaluated under light microscopy by a histopathologist.

Initially, tissue morphology was evaluated, and parameters such as the presence of fibrosis, necrosis, and inflammatory infiltrate were investigated, and then quantitative methods were used. For the liver, the fat deposition was calculated as the percentage of the area with fat in the hepatic tissue. Five random fields were evaluated per animal (20X objective).

For the evaluation of the duodenum, the length of the villi and the depth of the crypts were measured in 5 random fields per animal (10X objective), the villus:crypt ratio was used to analyze the area of intestinal absorption. Adipose tissue was evaluated in two areas of physiological deposits, subcutaneous and epididymal fat. For both, the area and diameter of adipocytes were quantified, as well as tissue morphology and the presence of inflammatory infiltrates. For the analysis, 5 random fields were used per animal (20X objective), or approximately 1,000 adipocytes were counted per animal.

Histopathological evaluations were performed using Axioscope Microscope and Camera Axiocam 208 color (Carl Zeiss Microscopy, Stockholm, Sweden), and quantifications using the Fiji/ImageJ Software and the Adiposoft plugin.

### Statistics

Data are presented as the mean ± SD unless otherwise indicated. Group comparisons were performed by one-way or two-way ANOVA, followed by post hoc Tukey's multiple comparisons test. Comparisons between two groups were performed using the unpaired t-test. A P value of less than 0.05 was considered statistically significant. The linear discriminant analysis effect size (LEfSe) method, including logarithmic discriminant analysis (LDA) scores and Kruskal–Wallis test, was used to compare the microbiota and identify taxa differing in relative abundance. The statistical analyses were performed using GraphPad Prism, version 9.2.0 (GraphPad Software), and SPSS v28 (IBM Corporation, Armonk, NY, USA).

## Results

### Chlorhexidine mouthwash induces a profound reduction in oral bacterial counts without interfering with food and water intake

**Figure 1:**
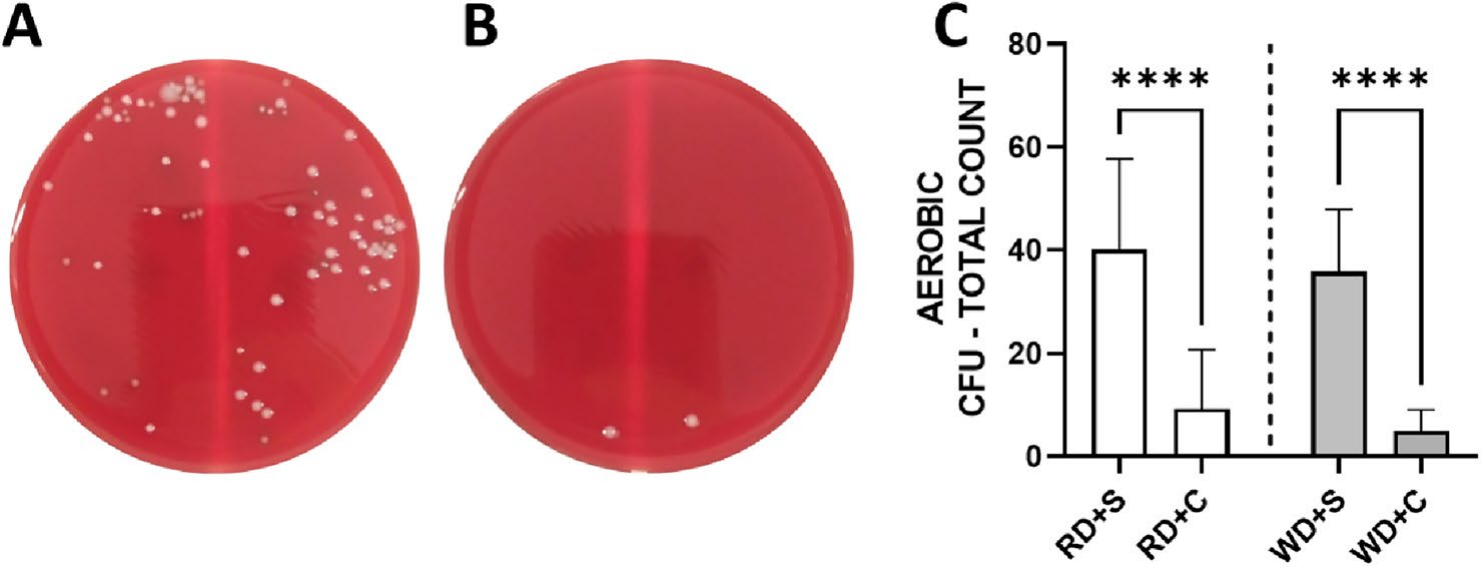
Total bacterial count of oral swab samples incubated for 18 h. Agar plate from an animal that received mouthwash with saline solution (**A**) and 0.2% chlorhexidine (**B**) after 8 weeks. Total bacterial count of the experimental groups at the end of the experimental protocol (**C**). Regular diet + saline (RD + S), RD + chlorhexidine (RD + C), Western diet + saline (WD + S), WD + chlorhexidine (WD + C). n = 12 mice in each experimental group. Data expressed in Mean ± SD.

In comparison to animals receiving mouthwash with saline solution, the chlorhexidine group had a reduction of more than 75% in the total count of viable bacteria in the oral cavity (Fig. 1A–C). The reduction was noted already after one week of chlorhexidine treatment, and it was similar regardless of dietary regime, i.e., regular diet (RD) or Western diet (WD) (Fig. 1C).

Water and food consumption remained constant throughout the experimental period in animals receiving RD. In the WD groups, likely due to the high caloric density (Supplementary material—Table S1–S2), consumption in grams was reduced after the first weeks, but there were no differences between the animals receiving chlorhexidine or saline mouthwash (Table 1). The effects of mouthwashes with isolated chlorhexidine or other components of the Corsodyl commercial formula, such as menthol and alcohol, were evaluated in pilot experiments and found not to affect eating behavior or water intake at the treatment frequency and concentration used here (Supplementary material—Figures S1–S2).

**Table 1:** Food and water intake.

	Regular diet			Western diet		
	Mouthwash saline	Mouthwash chlorhexidine	t-test *p*-value	Mouthwash saline	Mouthwash chlorhexidine	t-test *p*-value
Food Intake (g/mouse/day)	3.86 ± 0.08	3.67 ± 0.11	0.3903	3.19 ± 0.19	3.06 ± 0.14	0.9804
Calorie Intake (kcal/mouse/day)	17.18 ± 0.37	17.13 ± 0.51	0.3724	14.89 ± 0.88	14.29 ± 0.65	0.2958
Water Intake (ml/mouse/day)	4.19 ± 0.17	4.26 ± 0.32	0.9224	3.55 ± 0.30	3.73 ± 0.53	0.6488

Food and water consumption of mice fed with regular diet (RD) or Western diet (WD) high in fat and sugar for 8 weeks, and simultaneously received mouthwash with saline solution or 0.2% chlorhexidine. n = 12 mice in each experimental group. Data expressed in Mean ± SD.

### Chlorhexidine treatment is associated with lower weight gain and reduced fat accumulation in diet-induced obesity

In the mice receiving WD and chlorhexidine mouthwash for 8 weeks, a reduction in weight gain from the first week of treatment was observed. In line with this, these animals had less fat mass and percentage of body fat, as well as reduced epididymal fat compared to saline controls. Liver weight was significantly lower in the chlorhexidine group (Table 2). In contrast, chlorhexidine treatment of mice fed a regular diet did not cause any change in body weight gain or body composition, apart from a slightly lighter liver (Table 2).

**Table 2:** Body weight and composition by DEXA analysis.

	Regular diet			Western diet		
	Mouthwash saline	Mouthwash chlorhexidine	t-test *p*-value	Mouthwash saline	Mouthwash chlorhexidine	t-test *p*-value
Body weight (g)	31.74 ± 1.80	32.53 ± 2.47	0.3637	38.26 ± 3.62	34.99 ± 3.95	0.0374
BW gain (g)	0.87 ± 0.08	1.04 ± 0.12	0.2548	7.98 ± 2.06	5.50 ± 2.33	0.0157
Total mass (g)	31.38 ± 2.01	32.17 ± 2.25	0.4825	37.05 ± 3.41	33.46 ± 3.53	0.0219
Fat mass (g)	11.10 ± 1.73	11.35 ± 2.08	0.8437	13.75 ± 3.07	9.41 ± 2.96	0.0024
Lean mass (g)	19.35 ± 0.78	20.02 ± 1.16	0.2243	23.31 ± 1.85	24.04 ± 2.16	0.3748
BMC (g)	0.92 ± 0.06	0.87 ± 0.04	0.1382	0.52 ± 0.06	0.53 ± 0.03	0.7282
BMD (g/cm^2^)	0.08 ± 0.004	0.07 ± 0.003	0.1641	0.05 ± 0.002	0.05 ± 0.001	0.1785
Liver (g)	1.47 ± 0.15	1.30 ± 0.12	0.0406	1.97 ± 0.38	1.62 ± 0.23	0.0187
Epididymal Fat (g)	0.61 ± 0.27	0.54 ± 0.37	0.6983	2.39 ± 0.70	1.38 ± 0.54	0.0011

Body weight, weight gain, and organ weight data after 8 weeks of mouthwash with saline and chlorhexidine in mice fed with Regular or Western diet. (BW = Body weight; BMD = Bone mineral density; BMC = Bone mineral content). n = 12 mice in each experimental group. Data expressed in Mean ± SD.

**Figure 2:**
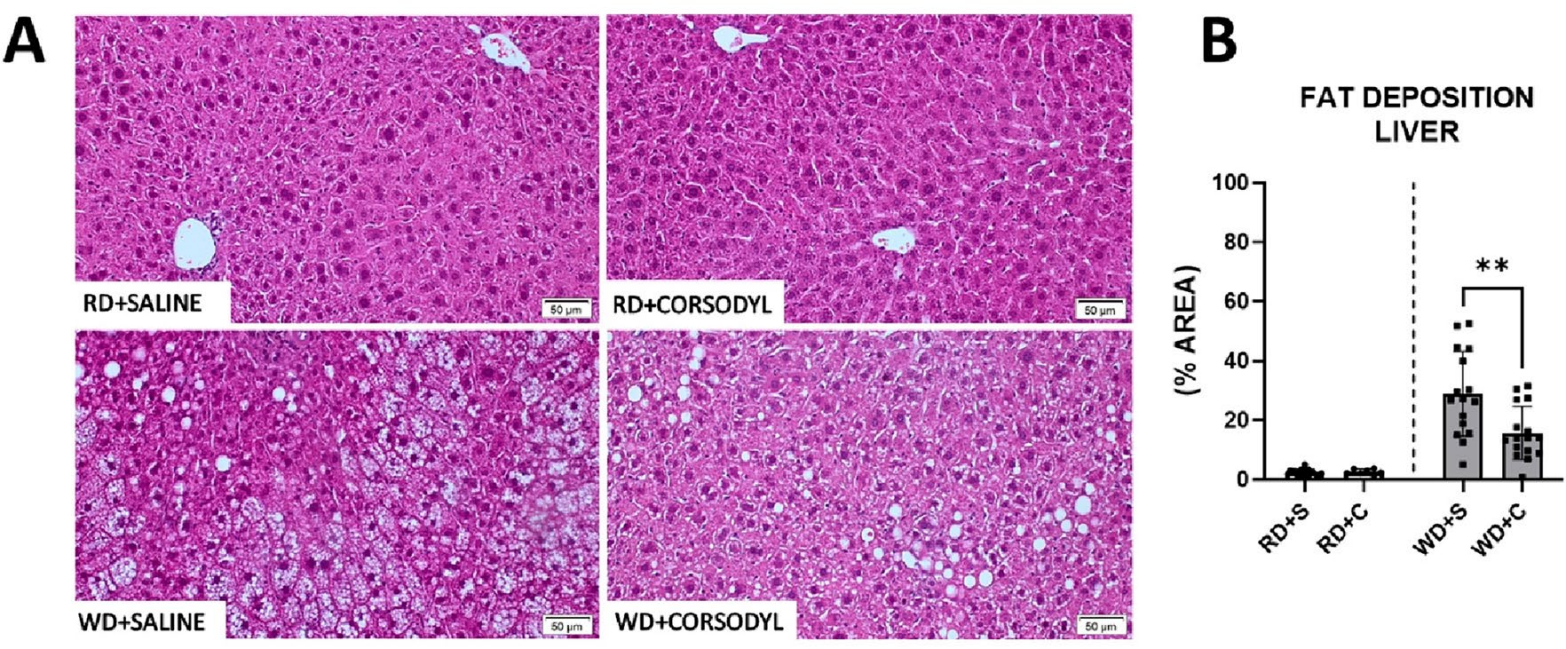
Histopathological evaluation of the liver of mice that received regular (RD) or Western diet (WD) for 8 weeks combined with saline- or chlorhexidine mouthwash. (**A**) Comparative panel with photomicrographs stained with hematoxylin–eosin showing the deposition of fat in the hepatocytes of the animals that consumed WD, and its absence in the groups with RD (20 × objective). (**B**) Quantification of the percentage of fat-filled area in hepatocytes and comparison between groups that received mouthwash for 8 weeks. Regular diet + saline (RD + S), RD + chlorhexidine (RD + C), Western diet (WD) + saline (WD + S), WD + chlorhexidine (WD + C). n = 12 mice in each experimental group. Data expressed in Mean ± SD. **Denotes *p* < 0.01.

Histopathological evaluation of hepatic fat deposition (Fig. 2A) showed that the WD animals exposed to chlorhexidine had less hepatic fat deposition than those exposed to saline. For the RD groups, liver tissue contained minimal fat deposition, and no difference was observed between chlorhexidine and saline (Fig. 2B).

### Effects of chlorhexidine mouthwash on glucose and insulin homeostasis, blood pressure and nitrate-nitrite levels

**Figure 3:**
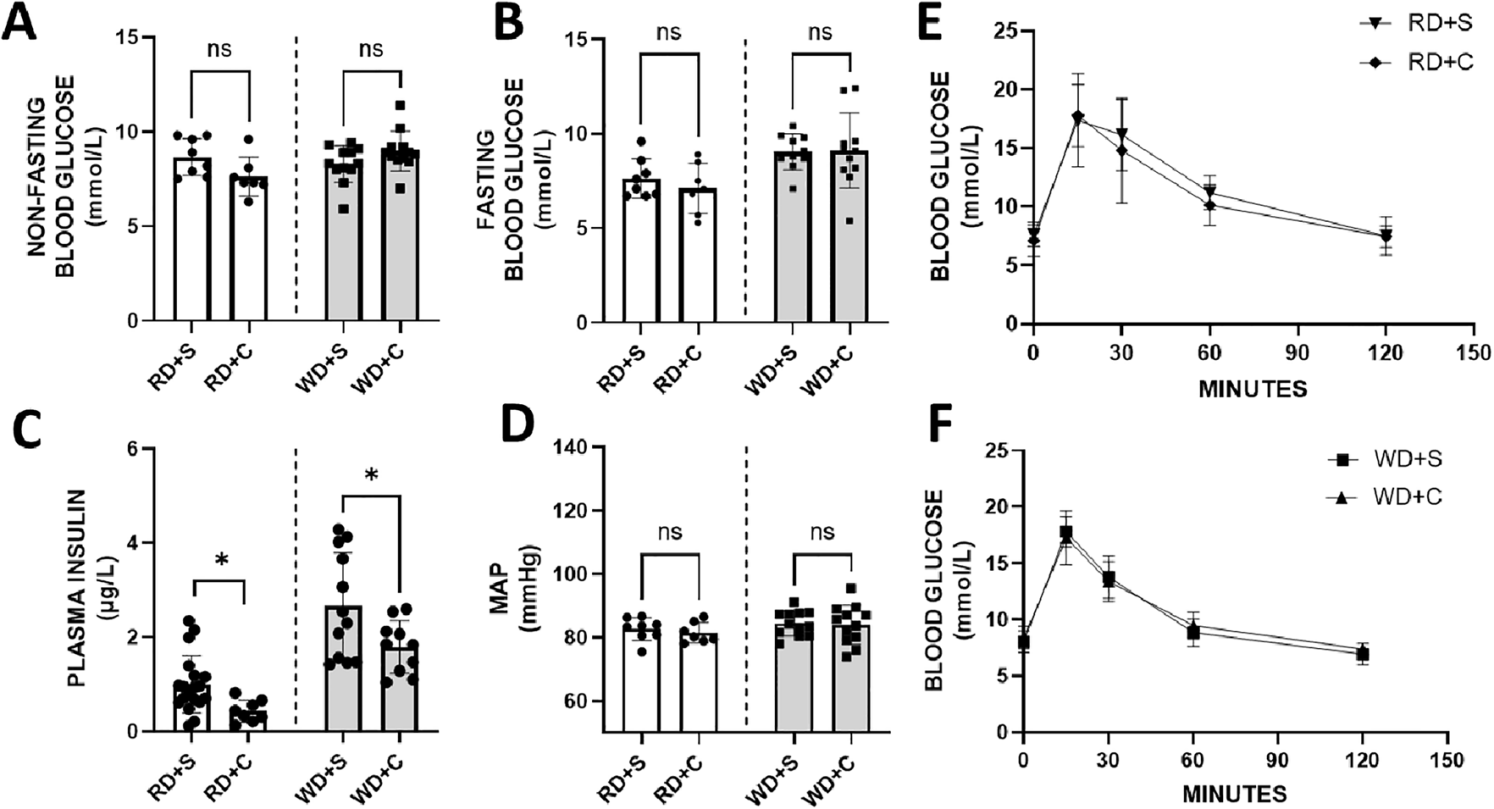
Metabolic tests of mice that received mouthwash with chlorhexidine or saline solution for 8 weeks. (**A**–**B**) Non-fasting and 5-h fasting plasma glucose. (**C**) Plasma insulin from samples collected in the early morning (approximately 8 a.m.). (**D**) Mean arterial pressure (MAP) collected in awake animals, using tail-cuff system, in the morning after 3 days of training. (**E**–**F**) Intraperitoneal glucose tolerance tests. Regular diet + saline (RD + S), RD + chlorhexidine (RD + C), Western diet (WD) + saline (WD + S), WD + chlorhexidine (WD + C). n = 12 mice in each experimental group. Data expressed in Mean ± SD. *Denotes *p* < 0.05.

Mice fed with WD had higher fasting glucose levels than those fed RD (*p* = 0.0011) (Fig. 3A–B) but with no impact from chlorhexidine exposure in any of the diet groups. In agreement, mice receiving WD had higher plasma insulin levels than RD-fed mice, but when WD-fed mice were treated with chlorhexidine, the insulin levels were reduced (Fig. 3C). In concert, this suggests a possible influence on glucose absorption and metabolism. However, the glucose (Fig. 3E–F) and insulin (Supplementary material—Figures S3) tolerance test outcomes were unaltered by chlorhexidine mouthwash irrespective of the dietary regime.

In humans, mouthwash with chlorhexidine can increase blood pressure, and this is associated with alterations in components of the nitrate-nitrite-NO pathway^28^. Here we found no effect of chlorhexidine on blood pressure regardless of diet regime (Fig. 3D), despite alterations in systemic levels of components of the nitrate-nitrite-NO pathway. Thus, RD fed animals receiving chlorhexidine mouthwashes had markedly lower plasma nitrite and RBC heme-NO levels than RD-fed, saline-treated animals (Table 3). The corresponding measures are not available for WD fed mice. The reason for these differences is presently unclear but might be related to the inability of rodents to concentrate nitrate in saliva^29^.

**Table 3:** Markers of nitric oxide metabolism.

	Regular diet			Western diet		
	Mouthwash saline	Mouthwash chlorhexidine	t-test *p*-value	Mouthwash saline	Mouthwash chlorhexidine	t-test *p*-value
Plasma Nitrate (µM)	31.05 ± 13.82	23.45 ± 9.61	0.2453	32.51 ± 6.51	32.88 ± 9.31	0.9044
Plasma Nitrite (µM)	1.51 ± 0.72	0.40 ± 0.20	0.0209	1.29 ± 0.50	1.46 ± 0.49	0.4508
Urinary Nitrate (µM)	368.5 ± 291.6	310.2 ± 229.1	0.6935	525.9 ± 124.9	488.5 ± 208.7	0.6160
Urinary Nitrite (µM)	1.60 ± 0.55	2.19 ± 0.89	0.1513	3.82 ± 1.97	2.84 ± 0.90	0.1349
RBC Heme-NO (a.u.)	10.56 ± 4.69	5.15 ± 3.10	0.0226	6.05 ± 1.90	4.77 ± 2.51	0.5688

Plasma and urinary concentrations of nitrate and nitrite, and heme-NO signal in blood, determined by electron paramagnetic resonance. n = 12 mice in each experimental group. Data expressed in Mean ± SD. Red blood cell (RBC), Arbitrary units (a.u.).

### Chlorhexidine mouthwash reduces macronutrient absorption without altering intestinal morphology

In WD fed mice, chlorhexidine mouthwash was associated with less weight gain and fat deposition though energy (calorie) intake remained unaltered (Table 4). These findings, together with the observed lower plasma insulin level, suggest that there may be alterations in the absorption of macronutrients. To investigate this, the levels of triglycerides and cholesterol in plasma and feces, and the total protein concentration in feces were evaluated. In line with the findings described above, WD-fed, chlorhexidine-exposed mice had higher concentrations of triglycerides and proteins in the feces than WD-fed unexposed mice (Table 4), supporting reduced absorption of these macronutrients. Fecal cholesterol levels were also numerically higher, although this did not reach statistical significance. No statistically significant difference was seen in the feces contents of RD-fed mice or in triglycerides or cholesterol levels in plasma in WD or RD fed mice (Table 4).

**Table 4:** Biochemical analysis of tissues.

	Regular diet			Western diet		
	Mouthwash saline	Mouthwash chlorhexidine	t-test *p*-value	Mouthwash saline	Mouthwash chlorhexidine	t-test *p*-value
Plasma Triglycerides (mg/dL)	54.64 ± 13.22	57.45 ± 20.63	0.7550	54.43 ± 11.18	49.84 ± 14.36	0.4200
Plasma Cholesterol (mg/dL)	55.70 ± 11.21	45.62 ± 6.18	0.0677	63.04 ± 22.69	73.20 ± 15.32	0.1215
Feces Triglycerides (mg/dL)	54.64 ± 13.22	76.37 ± 36.01	0.1346	231.6 ± 45.28	355.0 ± 88.13	0.0011
Feces Cholesterol (mg/dL)	63.94 ± 14.29	71.72 ± 18.08	0.3375	70.08 ± 16.08	75.28 ± 13.58	0.4225
Feces Protein (mg/dL)	146.3 ± 108.7	171.3 ± 96.74	0.6343	130.8 ± 56.38	175.7 ± 43.10	0.0429
Colon content Protein (mg/mL)	1.85 ± 0.65	1.96 ± 0.55	0.7211	0.83 ± 0.50	1.45 ± 0.43	0.0051
Lipase Activity Tongue (nmol/min/mL/mg protein)	0.014 ± 0.005	0.017 ± 0.008	0.3463	0.067 ± 0.023	0.054 ± 0.021	0.1854

Biochemical evaluation of plasma, colon content, feces and lipase activity in the tongue after 8 weeks of mouthwash with saline and chlorhexidine. n = 12 mice in each experimental group. Data expressed in Mean ± SD.

Lipase activity in the oral cavity was also investigated for its role in rodents' fat assimilation physiology. As expected, this was increased in mice fed WD compared to RD, but no significant effect of chlorhexidine mouthwash was observed in any of the diet groups (Table 4).

**Figure 4:**
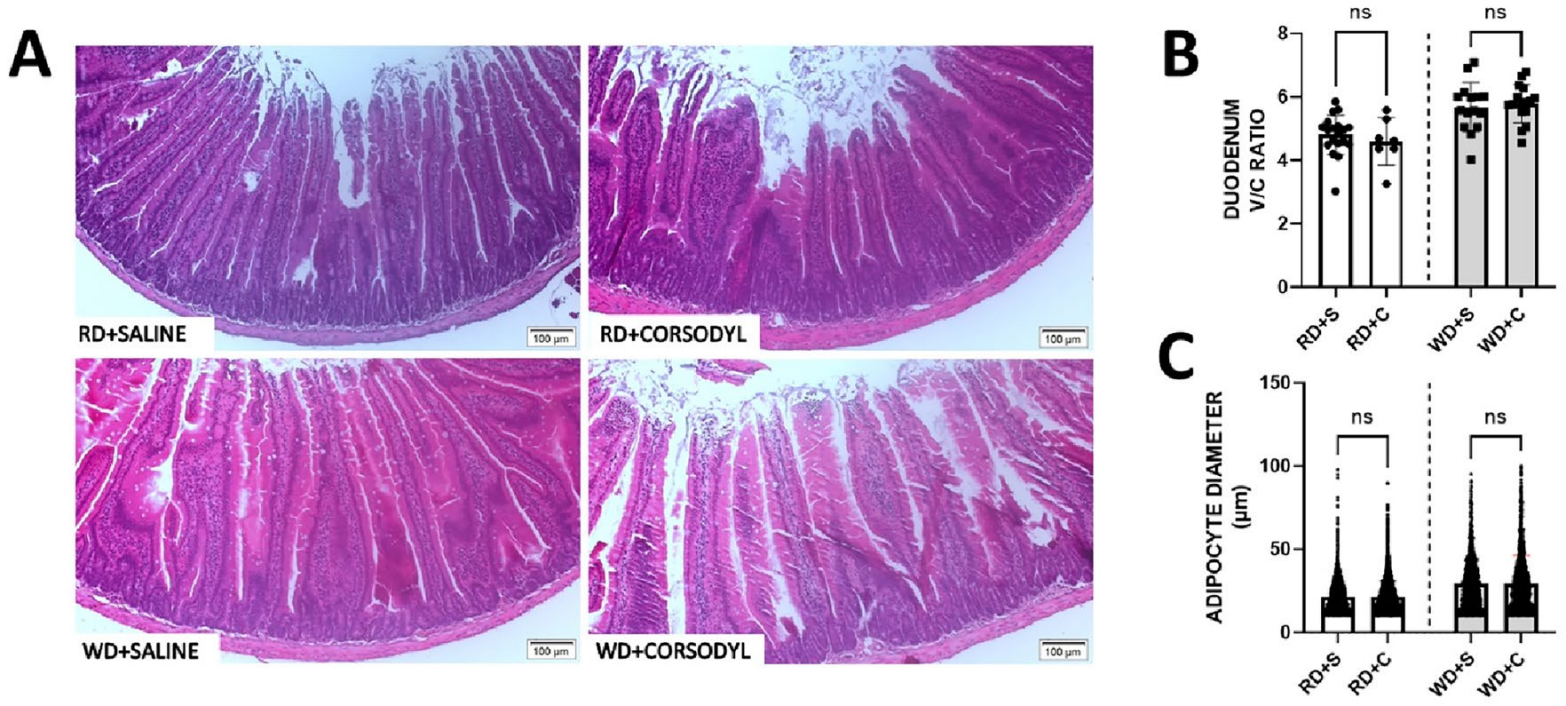
Histopathological evaluation of the duodenum of mice that received regular diet (RD) and Western diet (WD) for 8 weeks combined with saline and chlorhexidine mouthwash. (**A**) Comparative panel with photomicrographs stained with hematoxylin–eosin showing the villus length and the duodenal crypt length depth (10 × objective). (**B**) Quantification of the villi:crypt ratio and comparison between groups that received mouthwash for 8 weeks. (**C**) Quantification of adipocyte diameter (µm) in adipose tissue stored in the subcutaneous space of the right flank (20 × objective). Regular diet + saline (RD + S), RD + chlorhexidine (RD + C), Western diet (WD) + saline (WD + S), WD + chlorhexidine (WD + C). n = 12 mice in each experimental group. Data expressed in Mean ± SD.

Reduced absorption of nutrients may also be linked to a reduction in intestinal absorption. To exclude that this was the case in WD-fed, chlorhexidine-exposed mice, we performed a morphological evaluation of the duodenum. The area of absorption was quantified based on the ratio between the length of the villus and the depth of the duodenal crypt (V/C ratio) as shown in Fig. 4. The WD, which is rich in sugar and fat, caused an increase in duodenal villi length and in the V/C ratio (*p* < 0.0001) compared with RD, as has been described by others^30^. However, no differences were induced by the chlorhexidine mouthwashes (Fig. 4B). Similarly, fat deposits in the subcutaneous space of the duodenum the right flank, and gonadal fat, i.e., the area and diameter of the adipocytes, were higher in WD- compared with the RD-fed animals (*p* < 0.0001) regardless of chlorhexidine exposure (Fig. 4C, Supplementary material—Figure S4).

### Chlorhexidine mouthwash alters the gut microbiome

Given the consistent findings supporting an effect of chlorhexidine treatment on metabolic parameters in WD, but not RD-fed animals, the effect of chlorhexidine on the gut microbiota profile was evaluated in chlorhexidine-exposed WD-fed mice (n = 11) and with a WD saline-treated (n = 11) control and an RD-fed reference (n = 12) group. Hence, sequencing was done in DNA from 34 fecal samples as one mouse died, and one sample yielded insufficient DNA. In total, 2,586,490 quality-controlled sequences in 1,767 ASV features with an average (min, max) reads per sample of 34,487 (19,285, 61,352) were obtained. Of the 1,767 ASVs, 971 matched a named phylotype and 796 an unnamed phylotype. The latter were excluded from further analyses, and the former belonged to 69 genera, 36 families, 21 orders, 14 classes, and 7 phyla.

First, we evaluated that the Western diet induced a shift in the gut microbiota in line with what is reported in the literature. In brief, the feces microbiota in WD (saline exposed) versus RD-fed mice showed significantly lower α-diversity (lower observed richness (number of taxa) at all sequencing depths (Supplementary Figure S5A), and especially less abundance of taxa in genus *Lactobacillus* and enrichment in genus *Faecalibaculum* (Supplementary Figure S5B). Further, the two diet groups were distinctly separated based on Jaccard diversity in a PCoA plot (Supplementary Figure S5C). More details are shown in Supplementary Figure S5D–F.

**Figure 5:**
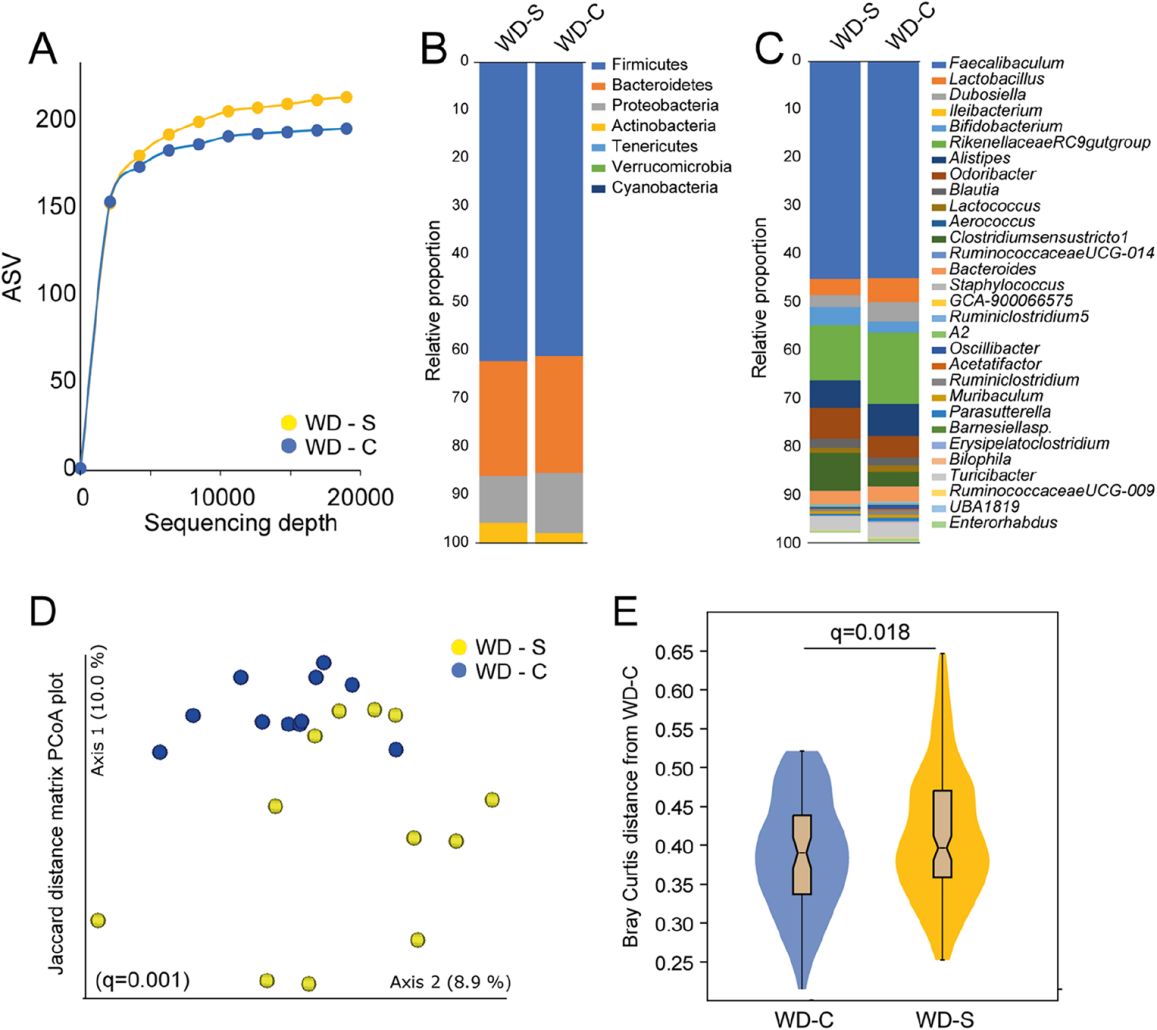
Effects of 0.2% chlorhexidine versus saline mouthwashes on the gut microbiota of mice fed a Western diet. The effects are shown for (**A**) rarefaction measured ASVs, relative abundance at the (**B**) phylum, and (**C**) genus level, (**D**) PCoA plot based on Jaccard distance, and (**E**) violin plots showing the distribution of Bray Curtis scores. Western diet + saline (WD-S, n = 11), WD + chlorhexidine (WD-C, n = 11).

**Figure 6:**
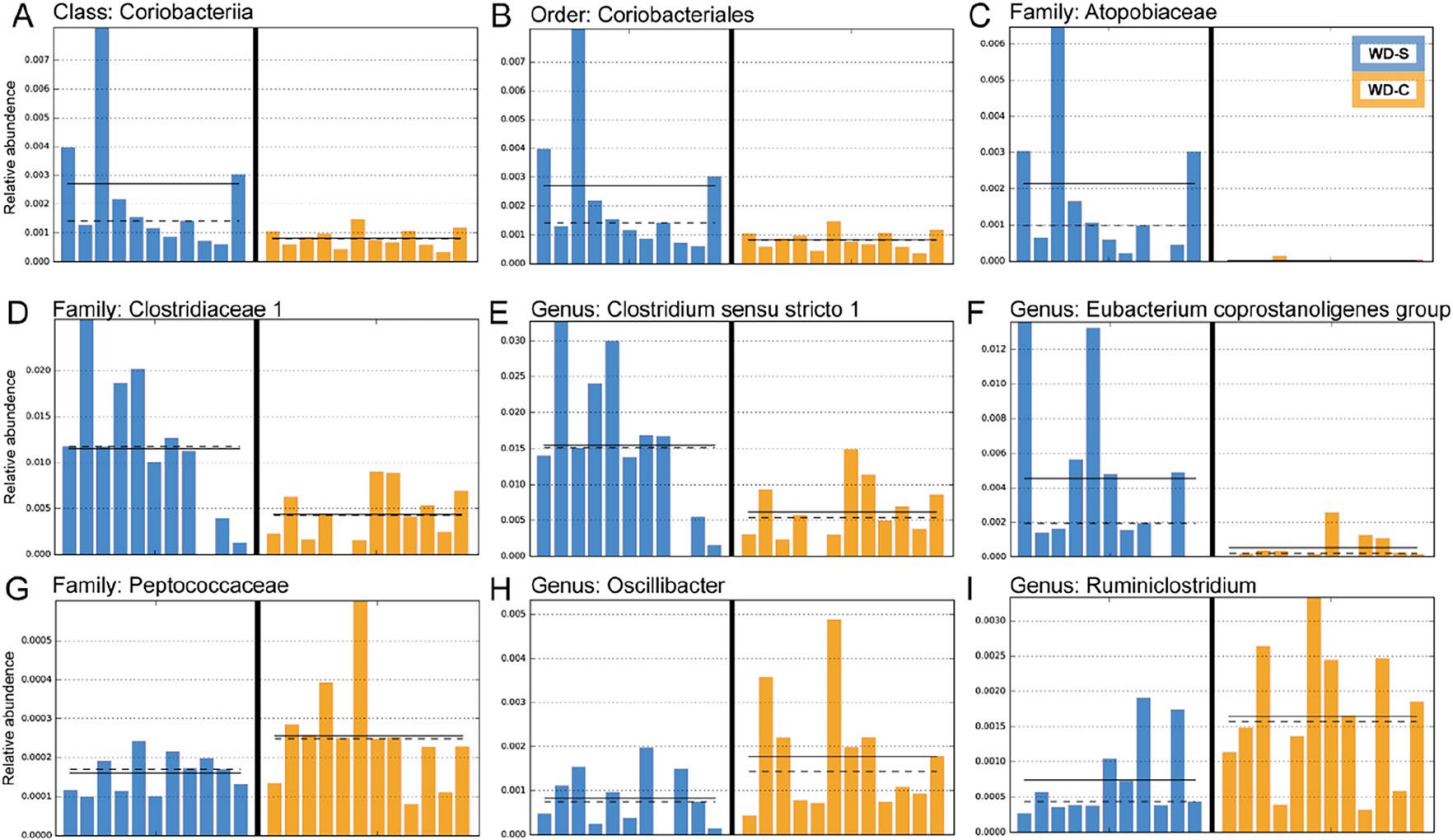
Panel of taxa with statistically significant difference in relative abundance in feces from mice fed a Western-type diet (WD) and mouth washings (three times/week for 8 weeks) with saline (WD-S) or 0.2% chlorhexidine (WD-C). The results are from Least discrimination Analysis (LDA) Effect Size (LEfSe) with significantly discriminative features set to an LDA score > 2.0.

Oral treatment with 0.5% chlorhexidine 3 times a week for 8 weeks in mice eating a high fat, high sugar, and low fiber diet (WD) tended to have lower species richness compared to that in treated with saline (Fig. 5A), with differences in relative abundance in several phyla and genera (Fig. 5B–C). This finding aligns with previously described research^31,32^. Further, the two groups were separated in a Jaccard distance matrix based PCoA plot (Fig. 5D) and differed significantly in Bray–Curtis distance matrix (Fig. 5E). Reductions in the gut microbiota of chlorhexidine versus saline-treated mice was particularly noted for taxa in the Coriobacteriia class and Coriobacteriales Order (Fig. 6A–B) which comprises genera like *Atopobium*, *Olsenella*, *Cryptobacterium*, and *Eggerthella*. These genera are known to be commonly present in the mouth too. Significant differences within the Coriobacteriia class were observed for the Coriobacteriaceae order, as well as the *Clostridiaceae 1* and *Atopobiaceae* families (Fig. 6C–D). Furthermore, WD-fed mice that received chlorhexidine mouthwashes exhibited lower relative abundances in the genera *Clostridium *sensu stricto* 1* and *Eubacterium coprostanoligenes* compared to those exposed to saline (Fig. 6E–F). Conversely, taxa in the *Peptococcaceae* family (Fig. 6G), as well as the genera *Oscillibacter* and *Ruminiclostridium* (Fig. 6H–I), showed higher relative abundances in the WD-fed mice treated with chlorhexidine.

## Discussion

In this study, we demonstrate that repeated topical application of a chlorhexidine antiseptic mouthwash in mice fed a high fat-high sugar-low fiber Western type diet led to significant alterations in the lower intestinal microbiome profile. This was associated with reduced macronutrient absorption, diminished overall weight gain, less fat accumulation in the liver, and decreased circulating insulin levels. Although these effects may seem beneficial in a model of diet-induced metabolic syndrome, it is important to consider that prolonged exposure could potentially lead to detrimental effects due to general malabsorption.

Current evidence suggests caution in prolonged antiseptic mouthwash use due to an association with cardiometabolic diseases^33,34^. Human studies show that chlorhexidine mouthwash can raise blood pressure and reduce NO-related metabolites (*i.e.* nitrate and nitrite)^34–36^. Conversely, we and others have shown favorable cardiometabolic effects of fueling the nitrate-nitrite-NO pathway by dietary nitrate in several models^12^. In this study, one may therefore reason that mouthwash's impact on the oral microbiome would negatively affect NO regulation and hence cardiometabolic features. However, we found no evidence of a significantly disturbed NO signaling in this study. However, It should be noted that the salivary nitrate concentrating ability is significantly lower in rodents than in humans^29^, which of course might influence the degree of impact on the nitrate-nitrite-NO pathway following antiseptic mouthwash using chlorhexidine.

In mice receiving regular diet (RD), we observed that chlorhexidine mouthwash significantly reduced plasma nitrite and RBC heme-NO (i.e*.*, an NO signaling entity in the vasculature)^37,38^. However, these effects were not observed in WD mice. Possibly, this could be explained by the lower nitrate content in the WD or that the high fat and sugar intake affected the ability of chlorhexidine to impact the oral microbiome. The lack of effects of chlorhexidine on NO metabolites during WD treatment, despite clear effects on the gut microbiome and nutrient handling, may suggest that other signaling entities play a more significant role.

In line with previous reports, major changes were induced in the gut microbiome by the introduction of a WD compared with a standard mice chow^31,39,40^. Interestingly, we also noted changes in the gut microbiome upon topical treatment with oral chlorhexidine. More specifically, bacteria belonging to the class *Coriobacteriia* and the families *Clostridiaceae* and *Atopobiaceae* were profoundly downregulated here by chlorhexidine mouthwash in WD-fed mice. At this stage, it is not possible to establish a direct link between these changes and the functional effects observed on fat and protein absorption. However, members of the families *Coriobacteriaceae* and *Clostridiaceae* are known to produce secondary bile acids, as well as to perform important metabolic functions, such as in the conversion of bile acids, steroids, and phytoestrogens^41^. Along the same line, *Atopobiaceae* bacteria have been reported to produce beneficial lactate and short-chain fatty acids^42^. In summary, the bacteria shown to be downregulated by the chlorhexidine mouthwash have been investigated earlier in the context of metabolic diseases, and their increased or decreased presence in the intestine has been correlated with different diseases such as gestational diabetes mellitus^43^, inflammatory bowel disease^44^, and obesity^45^.

The finding of increased triglycerides and proteins in intestinal content, combined with dysbiosis induced by chlorhexidine, suggests that chlorhexidine mouthwash may interfere with the absorption of macronutrients possibly through effects on the oral-intestinal microbiome axis^6^. Regarding fat metabolism, it is known that the intestinal microbiota can directly affect lipid metabolism and lipid levels in blood and tissues in mice and humans^46,47^. These effects are related to the production and diversity of bile acids, metabolism of short-chain fatty acids, production of lipopolysaccharides and effects on intestinal permeability^48^. In the present study, we cannot rule out that the gut may have been directly exposed to swallowed chlorhexidine, thereby affecting absorption. However, our duodenal histological evaluation did not reveal any macroscopical mucosal injury explaining the interference with the absorption of macronutrients. It is also possible that oral bacteria affect nutrient absorption by increasing metabolic efficiency, e.g., by partly digesting otherwise non-absorbable nutrients and converting them into absorbable energy. In such case, the mouthwash, which almost eliminated the oral microflora, could partly explain the reduced amount of absorbed nutrients.

We also identified a reduction of fermentative bacteria of the genus *Clostridium *sensu stricto* 1*, which are associated with the metabolism of various compounds such as carbohydrates, amino acids, alcohols, and purines with formation of butyric acid as a 'genus specific' product of fermentation^49^.

A considerable reduction in bacteria in the *Eubacterium coprostanoligenes* genus was observed in mice receiving mouthwash with chlorhexidine. These bacteria are known for cholesterol-to-coprostanol conversion in the colon and this gut bacterial metabolism has been linked to health and disease. Recent evidence suggests these bacteria could contribute to lower blood cholesterol and lower cardiovascular risk^50,51^. In human studies aiming to identify distinct gut microbial signatures in different levels of obesity, *Eubacterium coprostanoligenes* genus was suggested as a microbial biomarker of healthy people^52^.

Mouthwash with chlorhexidine was also responsible for increasing the presence of some bacteria in the intestine. This included the anaerobic gram-positive cocci of the family *Peptococcaceae*, known to be involved in various pathological conditions, such as arthritis^53^, endocarditis^54^, and brain abscess^55^. Thus, one might speculate that the increased presence of these bacteria combined with changes in the intestinal barrier can confer a greater risk of infection and systemic disorders in the long term.

The reduced absorption of fat and protein caused by mouthwash may seem overall beneficial in this experimental scenario, however, under healthy conditions or in the long term, harmful effects may be foreseen, and such possibilities need to be investigated, as well as effects in females and in older individuals.

## Conclusions

We conclude that prolonged use of chlorhexidine mouthwash in mice consuming a high fat-high sugar-low fiber Western type diet causes a disturbance in the gut microbiota profile, with an associated reduction in fat and protein absorption and an attenuated diet-induced obesity phenotype.

### Supplementary Information

Supplementary Information.

## Data Availability

The datasets used and/or analyzed during the current study are available from the corresponding author upon reasonable request.
