# Supplementary material for: Antibacterial mouthwash alters gut microbiome, reducing nutrient absorption and fat accumulation in Western diet-fed mice
Source: Sci Rep. 2024 Feb 18;14:4025. doi: 10.1038/s41598-024-54068-y (PMC10874955; doi:10.1038/s41598-024-54068-y)
Supplement: Supplementary file 1 — Supplementary Information. [file 41598_2024_54068_MOESM1_ESM.pdf]

Antibacterial mouthwash alters the gut microbiome and is associated with reduced nutrient absorption and less fat accumulation in mice fed a Western diet

Lucas Rannier Ribeiro Antonino Carvalho[1], Ariela M. Boeder[1,2], Miho Shimari[1], Andrei L. Kleschyov[1], Anders Esberg[3], Ingegerd Johansson[3], Eddie Weitzberg[1,4], Jon O Lundberg[1*#], Mattias Carlstrom[1*#]

[1] Department of Physiology and Pharmacology, Karolinska Institutet, Stockholm, Sweden
[2] Department of Pharmacology, Federal University of Santa Catarina, Florianópolis, Brazil
[3] Department of Odontology, Umeå University, Umeå, Sweden
[4] Department of Perioperative Medicine and Intensive Care, Karolinska Hospital, Stockholm, Sweden

* These authors contributed equally to this study.

# Corresponding author

Supplemental Figures:

Figure S1-S5

Supplemental Tables:

Table S1-S2

SUPPLEMENTARY MATERIAL

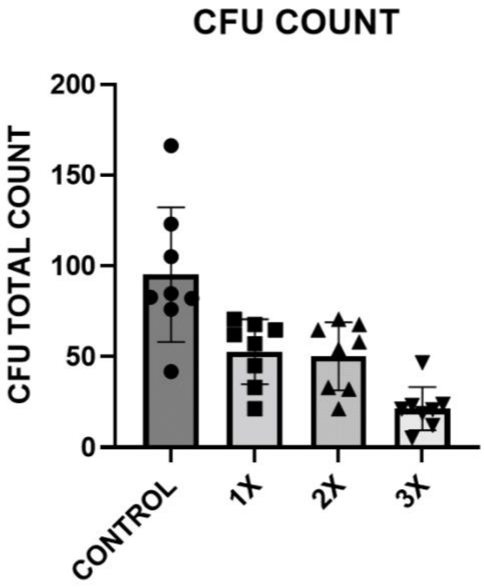

Figure S1*. Pilot evaluation of the frequency of mouthwashes with 0.2% chlorhexidine necessary to reduce the oral microbiome, evaluated by counting colony forming units (CFU) on blood agar plates. Average values from four weekly assessments. Samples collected using oral swabs of male mice (3 months old), mouthwashed with saline solution three times a week (control), chlorhexidine solution once a week (1x), twice a week (2x) and three times a week (3x). n=8 mice in each experimental group.*

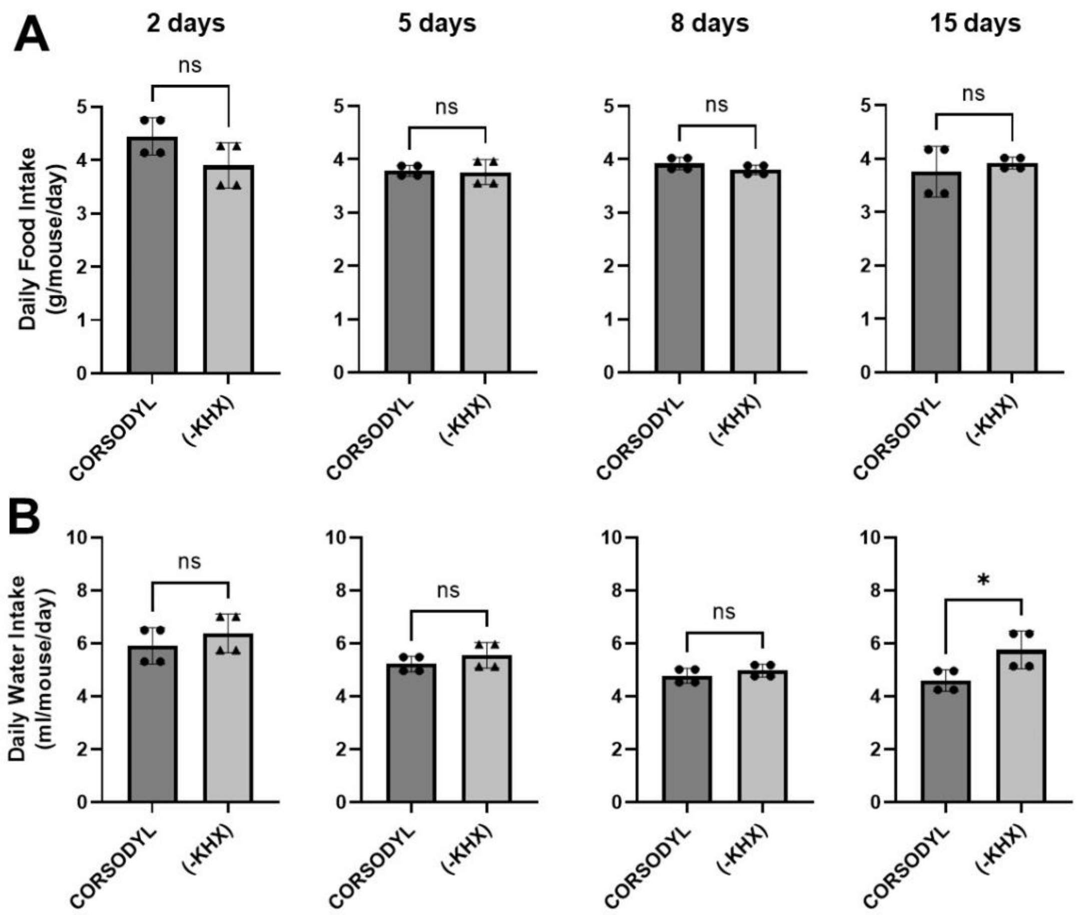

Figure S2. *Pilot evaluation of food (A) and water (B) intake for 15 days in mice subjected to mouthwash three times a week with commercial chlorhexidine solution (Corsodyl®), and homemade solution with the same components except chlorhexidine (-KHX). n=4 mice in each experimental group.*

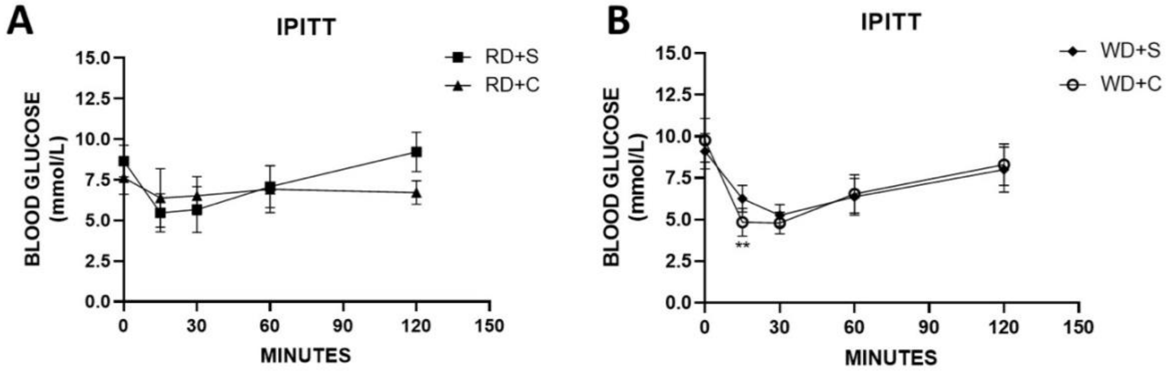

Figure S3. *Intraperitoneal insulin tolerance test (ipITT). In a fed state at the beginning of the day, the animals were challenged with an intraperitoneal dose of insulin, and serum glucose levels were monitored after 15, 30, 60 and 120 minutes of the administration. No statistical differences were observed between the groups that consumed a regular diet (A) or Western diet (B) and received mouthwash with saline or chlorhexidine. n=12 mice in each experimental group.*

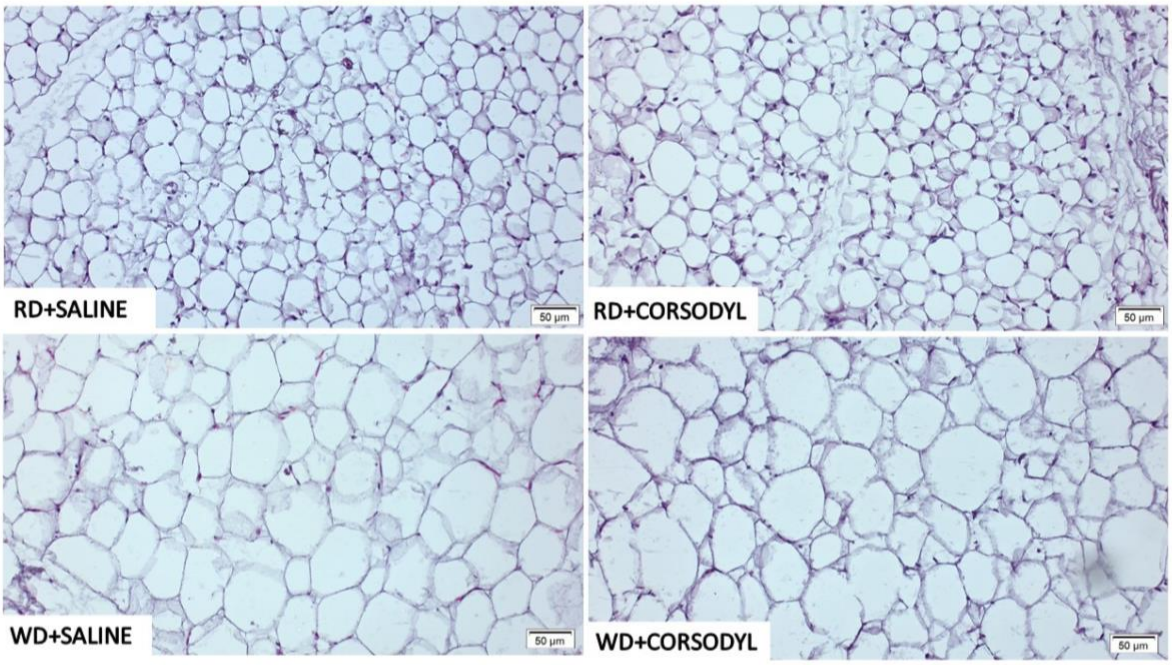

Figure S4. *Histopathological evaluation of the adipose tissue (subcutaneous fat sample) of mice that received regular (RD) or Western diet (WD) for 8 weeks combined with saline- or chlorhexidine mouthwash. Comparative panel with photomicrographs stained with hematoxylin-eosin showing a larger average size of adipocytes in the groups that consumed the Western diet, but without differences regarding exposure to mouthwash with chlorhexidine (20x objective).*

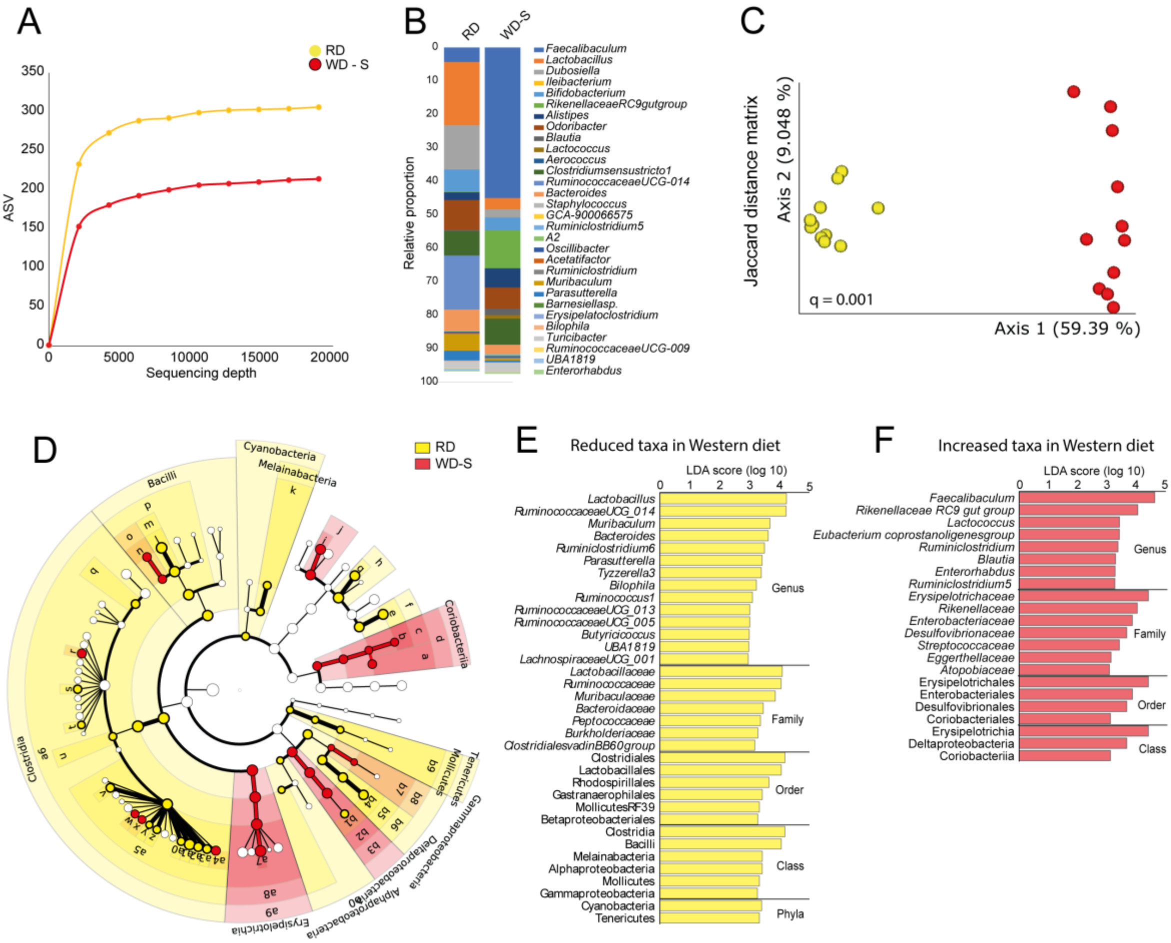

Figure S5. *Effects of the diet regimen on the GI microbiota. Feces samples were collected and compared from mice fed regular chow (RD) or Western diet and saline (WD-S) for 8 weeks. (A) Rarefaction measured ASV, (B) genus level presented as the average proportions and (C) Jaccard distance matrix for the groups with RD and WD. (D) Representative commensal intestinal microbial community of animals on the regular and Western diets, and (E-F) description of bacterial groups that suffered reduction or increase caused by the consumption of a high fat-high sugar-low fiber Western-type diet.*

Table S1. *Formulation of the Research Diet Western Diet (ca nº D12079B) used in the experiment to evaluate the effects of mouthwash with chlorhexidine in mice. Data available at: https://researchdiets.com/en/formulas/d12079b*

Class description	Ingredients	Grams
Protein	Casein, Lactic, 30 Mesh	195.00 g
Protein	Methionine, DL	3.00 g
Carbohydrate	Sucrose, Fine Granulated	350.00 g
Carbohydrate	Lodex 10	100.00 g
Carbohydrate	Starch, Corn	50.00 g
Fiber	Solka Floc, FCC200	50.00 g
Fat	Butter, Anhydrous (Discontinued)	200.00 g
Fat	Corn Oil	10.00 g
Mineral	S10001A	17.50 g
Mineral	Calcium Phosphate, Dibasic	17.50 g
Mineral	Calcium Carbonate, Light, USP	4.00 g
Vitamin	Choline Bitartrate	2.00 g
Vitamin	V10001C	1.00 g
Anti-oxidant	Ethoxyquin	0.04 g
Special	Cholesterol, NF	1.50 g
**Total:**		**1001.54 g**

Table S2. *Caloric information of the Research Diet Western Diet (Cat No. D12079B) used in the experiment to evaluate the effects of mouthwash with chlorhexidine in mice. Data available at: https://researchdiets.com/en/formulas/d12079b*

**Caloric information**	**D12079B Western Diet**
Protein	17% kcal
Fat	40% kcal
Carbohydrate	43% kcal
Energy density	4.67 kcal/g
